# Supplementary material for: Co-infection with Streptococcus and Rothia spp. drives prophage dynamics in Pseudomonas aeruginosa in an artificial sputum model
Source: ISME Commun. 2026 Jul 14;6(1):ycag191. doi: 10.1093/ismeco/ycag191 (PMC13600431; doi:10.1093/ismeco/ycag191)
Supplement: Supplementary file 1 [file supplementary_material_ycag191.zip › RNAseq_paper_supplements_v3.docx]

**Supplementary Figure 1.** Prophage 1: Pyocin R2. Log2FC values distribution of LESB58 prophage genes during inter-species bacterial interactions.

**Supplementary Figure 2.** Prophage 2: F10-like. Log2FC values distribution of LESB58 prophage genes during inter-species bacterial interactions.

**Supplementary Figure 3.** Prophage 3: F10-like. Log2FC values distribution of LESB58 prophage genes during inter-species bacterial interactions.

**Supplementary Figure 4.** Prophage 5: D3-like. Log2FC values distribution of LESB58 prophage genes during inter-species bacterial interactions.
